# A Role for Genetic Modifiers in Tubulointerstitial Kidney Diseases

**DOI:** 10.3390/genes14081582

**Published:** 2023-08-03

**Authors:** Gary P. Leggatt, Eleanor G. Seaby, Kristin Veighey, Christine Gast, Rodney D. Gilbert, Sarah Ennis

**Affiliations:** 1 Human Genetics & Genomic Medicine, University of Southampton, Southampton SO16 6YD, UK; egs1g09@soton.ac.uk (E.G.S.); k.veighey@soton.ac.uk (K.V.); christine.gast@porthosp.nhs.uk (C.G.); rodney.gilbert@uhs.nhs.uk (R.D.G.); se@soton.ac.uk (S.E.); 2 Wessex Kidney Centre, Queen Alexandra Hospital, Portsmouth Hospitals NHS Trust, Portsmouth PO6 3LY, UK; 3 Renal Department, University Hospital Southampton, Southampton SO16 6YD, UK; 4 Department of Paediatric Nephrology, Southampton Children’s Hospital, University Hospital Southampton NHS Foundation Trust, Southampton SO16 6YD, UK

**Keywords:** tubulointerstitial kidney disease, genetic modifiers, modifier genes, monogenic TKD, ADTKD

## Abstract

With the increased availability of genomic sequencing technologies, the molecular bases for kidney diseases such as nephronophthisis and mitochondrially inherited and autosomal-dominant tubulointerstitial kidney diseases (ADTKD) has become increasingly apparent. These tubulointerstitial kidney diseases (TKD) are monogenic diseases of the tubulointerstitium and result in interstitial fibrosis and tubular atrophy (IF/TA). However, monogenic inheritance alone does not adequately explain the highly variable onset of kidney failure and extra-renal manifestations. Phenotypes vary considerably between individuals harbouring the same pathogenic variant in the same putative monogenic gene, even within families sharing common environmental factors. While the extreme end of the disease spectrum may have dramatic syndromic manifestations typically diagnosed in childhood, many patients present a more subtle phenotype with little to differentiate them from many other common forms of non-proteinuric chronic kidney disease (CKD). This review summarises the expanding repertoire of genes underpinning TKD and their known phenotypic manifestations. Furthermore, we collate the growing evidence for a role of modifier genes and discuss the extent to which these data bridge the historical gap between apparently rare monogenic TKD and polygenic non-proteinuric CKD (excluding polycystic kidney disease).

## 1. Introduction

**Figure 1:**
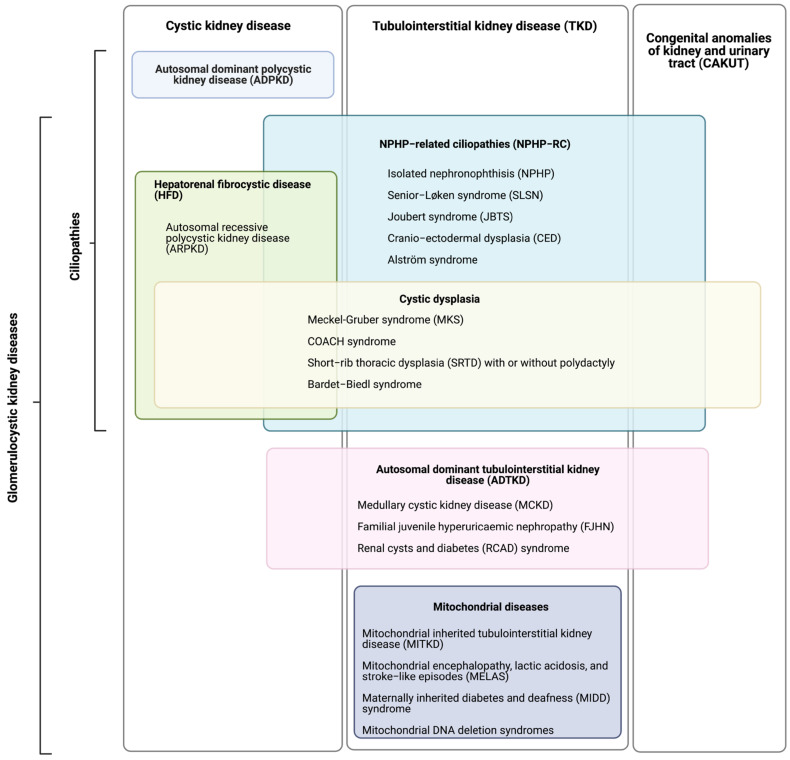
Synonymous and umbrella terms in tubulointerstitial kidney disease (TKD).

Tubulointerstitial kidney diseases (TKD) primarily involve the renal interstitium and tubular compartments. This typically results in tubulointerstitial fibrosis and tubular atrophy (IF/TA). The commonest TKDs include nephronophthisis and mitochondrially inherited, and autosomal-dominant tubulointerstitial kidney diseases (ADTKD). TKD results from a growing number of single-gene (monogenic) disorders. These are clinically characterised by a progressive decline in kidney function, leading to chronic kidney disease (CKD) and end-stage kidney disease (ESKD). TKD typically results in non-proteinuric CKD and is clinically differentiated from glomerular diseases by a lack of glomerular proteinuria and haematuria. However, more significant proteinuria can occur in the late stages due to secondary glomerulosclerosis. There may be impaired urinary concentrating ability and sodium reabsorption, resulting in polyuria and polydipsia. Extra-renal manifestations are highly variable (Table 1). In some instances, there may be renal cysts or structural abnormalities overlapping with polycystic kidney diseases (PKD) and congenital anomalies of the kidney and urinary tract (CAKUT), which are outside the scope of this review (Figure 1).

Initially “lumped” under the term “nephronophthisis-medullary cystic kidney disease” complex, the availability of genomic sequencing technologies has led to more detailed phenotypic and molecular characterisation. However, genetic and phenotypic nomenclature are now used interchangeably, creating the potential for confusion within multidisciplinary teams comprising geneticists and nephrologists. As patients have received genetic diagnoses, it has become clear that older terms such as familial juvenile hyperuricaemic nephropathy (FJHN) and medullary cystic kidney disease (MCKD) have needed updating (Figure 1 and Table 1). Multiple investigators have since demonstrated that neither tubular microcysts nor larger cysts are pathognomonic for these diseases, nor does the medulla appear to be the specific location for cysts when occasionally observed [1,2,3,4]. Some cases may be associated with hyperuricaemia and gout, but this is not pathognomonic and may be clinically silent in early disease [2,5]. In an attempt to rectify this, the term ADTKD has been suggested by international guidelines, with the term ADTKD-NOS where the causal gene is unknown [6]. Renal cysts and diabetes (RCAD) describes one of the potential manifestations of *HNF1B* variants that includes isolated TKD and congenital anomalies of the kidney and urinary tract (CAKUT).

Nephronophthisis is characterised by IF/TA, tubular basement membrane abnormalities, and cystic dilatation of the collecting duct [7]. Extra-renal manifestations, including several eponymous syndromes (Table 1), are seen in approximately one-fifth of nephronophthisis cases and are collectively known as NPHP-related ciliopathies (NPHP-RC) [8,9,10]. A subset of ciliopathies recently named hepatorenal fibrocystic disease is characterised not only by renal IF/TA (with or without cysts) but also fibrosis and cystic dysgenesis of the liver and porto-biliary tract [11]. Hepatorenal fibrocystic disease also includes autosomal-recessive polycystic kidney disease (ARPKD), characterised by congenital hepatic fibrosis and cystic dilatation of the renal collecting duct and most commonly resulting from variants in the ciliary IPT domain-containing fibrocystin/polyductin (*PKHD1*) [12]. Autosomal-dominant polycystic kidney disease (ADPKD) caused primarily by variants in polycystins 1 (*PKD1*) and 2 (PKD2) is also a ciliopathy; however, along with ARPKD, it falls outside the scope of this review, as cysts are the principal feature. Cystic dysplastic kidneys may occur alongside tubulointerstitial fibrosis in the lethal multisystem Meckel–Gruber syndrome. Cystic dysplasia or IF/TA may be present in Bardet–Biedl syndrome, short-rib thoracic dysplasia, and COACH syndrome (Table 1).

Mitochondrial cytopathies result from inherited or sporadic variants in mitochondrial DNA (mtDNA) or nuclear DNA that affect mitochondrial function and may present with nephrotic syndrome, Fanconi syndrome, TKD alone, or multisystem disorders [13,14,15]. It has been suggested that the term mitochondrially inherited tubulointerstitial kidney disease (MITKD) is used to complement ADTKD [14].

Most autosomal-recessive TKD results from loss-of-function variants in genes encoding non-motile (primary) ciliary proteins. Cilia are organelles projecting from most mammalian cell types that communicate signals from the extracellular environment and other cells. Ciliopathies can result in isolated TKD, known as nephronophthisis (from the Greek for wasting of the nephron), as well as multisystem diseases (Figure 1 and Table 1). Over thirty genes are associated with nephronophthisis (Table 2); nephrocystin-1 (*NPHP1*) accounts for 20% of cases and other genes < 3% each [16]. ADTKD is caused by heterozygous variants in any one of at least five genes, including *UMOD*, *MUC1*, *HNF1B*, *REN*, and *SEC61A1* (Table 2). The best-studied is the *UMOD* gene encoding uromodulin, the most abundant protein in human urine [17]. Recently, a phenotype with features of both TKD and PKD was attributed to variants in *DNAJB11* [18]. Mitochondrially inherited TKD is also well described [13].

**Figure 2:**
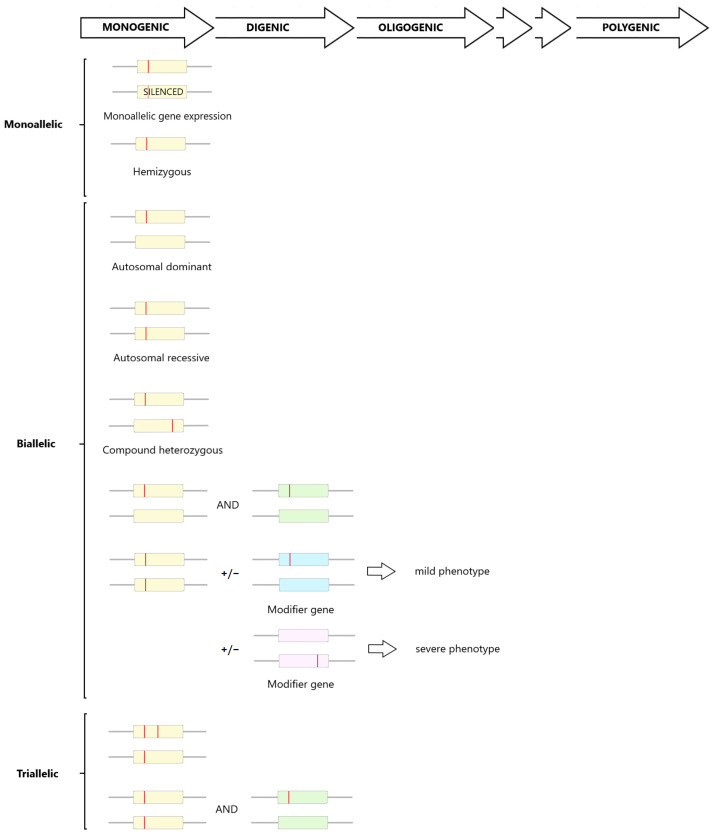
Simplified schematic representation of complex genetic inheritance from monogenic to polygenic (boxes represent genes; red vertical lines represent the pathogenic allele). A spectrum of increasingly complex diseases may occur with additional mutated alleles from monoallelic through to multiallelic. Additional complexity arises due to the number of genes affected from one gene (monogenic), a few genes (oligogenic), or many genes (polygenic). Modifiers are variants that can alter the disease phenotype but are not required for the primary disease to be present. Modifiers can be additive or suppressive and can affect penetrance, expressivity, and dominance.

Genetic TKD lies along a spectrum ranging from dramatic syndromic manifestations, typically diagnosed in childhood, to a more subtle phenotype difficult to distinguish from common forms of non-proteinuric CKD. Divergent phenotypes may be explained by variants of differing impact to gene functionality or mutated protein abundance. For example, homozygous variants in *TMEM231*, ranging from missense to null, are associated with diverse phenotypes, including Meckel–Gruber syndrome (MKS), orofaciodigital syndrome (OFD) type 3, and Joubert syndrome (JBTS) (Table 1) [19,20,21]. Furthermore, variants in constrained gene regions are more likely to impact protein function exemplified by heterozygous variants in the EGF2, EGF3, and D8C domains of *UMOD*, resulting in earlier-onset ESKD [5,22]. However, patients with the same primary pathogenic variant often manifest significant differences in penetrance or expressivity [23]. The wide variation in age of onset of CKD and extra-renal manifestations, including distinct syndromic disorders, is not adequately explained by our understanding of single-gene disorders alone. This brings into question the historical dichotomy of kidney diseases as either monogenic or polygenic (Figure 2) [5,8,24,25,26]. Whilst tissue mosaicism has been demonstrated in ADPKD and Alports, there is no evidence for this in TKD, which suggests that mosaicism is not modifying variable expressivity [27].

Monogenic forms result from rare variants with large effect sizes often manifesting severe disease (but that may show variable penetrance), whereas polygenic forms result from the cumulative effect of multiple common variants, causing relatively milder disease. However, recent data suggest that monogenic disease risk may vary substantially due to polygenic background [28,29]. Common variants in genes known to cause rare monogenic diseases are also associated with markers of CKD (e.g., *UMOD, MUC1*, *IQCB1* (*NPHP5*), *SDCCAG8*, and *IFT172*) [30,31,32]. This is further supported by gene-burden-association tests on 450,000 UKBioBank whole exomes, available at genebass.org, showing that *UMOD*, *PKD2*, and *SLC22A2* are most statistically associated with CKD [33].

The concept of genetic modifiers was first introduced in 1941 by Haldane, but multiple definitions have since been suggested [34]. Herein, modifier genes are defined as genes that alter the disease phenotype but are not required for the primary disease to be present. This is distinct from digenic or oligogenic inheritance, whereby two or more genes are essential for the manifestation of the primary disease phenotype [35]. Genetic modifiers can affect the expression of another gene at multiple different organisational levels, including transcription, protein interactions, or the cellular and organ level [36]. Terms such as epistasis, digenic/oligogenic inheritance, or modifier genes are often used interchangeably to describe the effect of one gene/allele on the phenotypic outcome of another gene/allele [35]. For tri-allelic inheritance (which can be synonymous with digenic or oligogenic inheritance), at least three mutated alleles are required to manifest a primary disease phenotype. Most typically, this involves both alleles of one gene and at least one allele in a second gene; however, all three variants can occur on the same gene (Figure 2) [37]. Modifiers can be additive or suppressive and can affect penetrance, expressivity, and dominance. This review collates the evidence for the role of modifier genes and oligogenic inheritance on the phenotypic spectrum of TKD and attempts to bridge the gap between apparently rare monogenic TKD and polygenic non-proteinuric CKD. Whilst epigenetic, environmental, and non-coding factors may also play a role, these are outside the scope of this review.

### 1.1. The Evidence for Modifier Genes

Modifier effects may be collectively common but are likely to be individually rare and heterogeneous and have therefore largely eluded discovery in underpowered studies. Most evidence comes from candidate gene or pathway approaches, with the more common variants likely to be tested first. Evidence for rare modifiers comes primarily from family case studies and are shown in Table 2.

**Table 2:** Monogenic causes of TKD and evidence of modifier genes.

HGNC Gene Symbol	HGNC-Approved Gene Name	Diagnosis	Gene Location	Assumed Inheritance Pattern	Oligogenic/Modifier Genes
Nephronophthisis					
*NPHP1* [38,39]	Nephrocystin 1	Nephronophthisis 1; Senior–Løken syndrome 1; Joubert syndrome 4; Bardet–Biedl syndrome.	2q13	AR	Homozygous *NPHP1* is possibly modified by heterozygous *NPHP4* with early-onset ESKD [40]; Heterozygous *AHI1* variants are enriched in patients with homozygous *NPHP1* and neurological symptoms [41].
*NPHP3* [42]	Nephrocystin 3	Nephronophthisis 3; Senior–Løken syndrome 3; Meckel–Gruber syndrome 7; Renal-hepatic-pancreatic dysplasia 1; Situs inversus; Hepatic fibrosis.	3q22.1	AR	Possible digenic inheritance with *INVS* [40]; A heterozygous *NPHP4* variant may modify compound heterozygous *NPHP3* with early-onset ESKD and hepatic fibrosis [40,43]; In patients with syndromic nephronophthisis caused by several genes (including *NPHP3*, *IQCB1*, *CEP290*, and *MKS1*), an additional heterozygous variant in *RPGRIP1L* is associated with retinitis pigmentosa [44].
*CEP290* [45,46]	Centrosomal protein 290	Nephronophthisis 6; Senior–Løken syndrome 6; Joubert syndrome; Bardet–Biedl syndrome 14; Hepatic fibrosis; Meckel–Gruber syndrome 4.	12q21.32	AR	Heterozygous pathogenic variants are present in several cases of homozygous *NPHP1* but with no evidence of modifier effect on phenotype [41]; There is a more severe neurological disease in a patient with bi-allelic *CEP290* variants and a heterozygous *AHI1* [47]; Possible tri-allelic disease with heterozygous *TMEM67* and homozygous *CEP290* variants in BBS [48]; In patients with syndromic nephronophthisis caused by several genes (including *NPHP3*, *IQCB1*, *CEP290*, and *MKS1*), an additional heterozygous variant in *RPGRIP1L* is associated with retinitis pigmentosa [44]; A variant in barttin CLCNK-type accessory subunit beta (*BSND*) I, significantly associated with kidney disease severity in patients with CEP290 variants [49].
*RPGRIP1L* [50]	Retinitis pigmentosa; GTPase regulator-interacting protein 1 like protein.	Nephronophthisis 8; Joubert syndrome 7; COACH syndrome; Hepatic fibrosis; Meckel–Gruber syndrome 5.	16q12.2	AR	In patients with syndromic nephronophthisis caused by several genes (including *NPHP3*, *IQCB1*, *CEP290*, and *MKS1*), an additional heterozygous variant in *RPGRIP1L* is associated with retinitis pigmentosa [44].
*TTC21B* [51]	Tetratricopeptide repeat domain 21B	Nephronophthisis 12; Joubert syndrome 11; Short-rib thoracic dysplasia; Focal segmental glomerulosclerosis (FSGS).	2q24.3	AR, AD	Both causal and a possible modifier of multiple ciliopathy genes. including *BBS1*, *BBS2*, *BBS4*, *MKKS*, *BBS7*, *BBS10*, *BBS12*, *NPHP4*, *CC2D2A,* and *TMEM216* [51]; *TTC21B* is a possible modifier in patients with FSGS and collagen type 4 gene variants (*COL4A3* and *COL4A5*) [52].
*INVS* [53]	Inversin	Nephronophthisis 2; Situs inversus; Hepatic fibrosis.	9q31.1	AR	Possible digenic inheritance with *NPHP3* [40].
*NPHP4* [54,55]	Nephrocystin 4	Nephronophthisis 4; Senior–Løken syndrome 4; Hepatic fibrosis.	1p36.1	AR	Homozygous *NPHP1* possibly modified by heterozygous *NPHP4* with early-onset ESKD [40]; Compound heterozygous *NPHP3* modified by heterozygous *NPHP4* variant with early-onset ESKD and hepatic fibrosis [40,43]; *TTC21B* contributes possible modifier alleles to *NPHP4* [51].
*IQCB1* [56]	IQ motif-containing B1	Nephronophthisis 5; Senior–Løken syndrome 5.	3q13.33	AR	Common variants are associated with elevated creatine in association studies [32]; In patients with syndromic nephronophthisis caused by several genes (including *NPHP3*, *IQCB1*, *CEP290*, and *MKS1*), an additional heterozygous variant in *RPGRIP1L* is associated with retinitis pigmentosa [44].
*GLIS2* [57]	GLIS family zinc finger 2	Nephronophthisis 7	16p13.3	AR	
*NEK8* [58]	NIMA-related kinase 8	Nephronophthisis 9; Renal-hepatic-pancreatic dysplasia 2; Hepatic fibrosis.	17q11.2	AR	
*SDCCAG8* [59]	Serologically defined colon cancer antigen 8	Nephronophthisis 10; Senior–Løken syndrome 7; Bardet–Biedl syndrome 16; Intellectual disability.	1q43-q44	AR	Common variants in *SDCCAG8* are associated with elevated creatine in association studies [32].
*TMEM67* [60]	Transmembrane protein 67	Nephronophthisis 11; Joubert syndrome 6; Meckel–Gruber syndrome 3; COACH syndrome; Hepatic fibrosis.	8q22.1	AR	Possible tri-allelic disease in BBS contributing heterozygous *TMEM67* variants to homozygous truncating variants in *CEP290* [48]; Possible tri-allelic disease contributing heterozygous *TMEM67* and homozygous *BBS9* variants in BBS [48].
*WDR19* [61]	WD repeat domain 19	Nephronophthisis 13; Senior–Løken syndrome 8; Cranio-ectodermal dysplasia 4; Short-rib thoracic dysplasia 5; Hepatic fibrosis.	4p14	AR	
*ZNF423* [62]	Zinc finger protein 423	Nephronophthisis 14; Joubert syndrome 19; Situs inversus.	16q12.1	AR, AD	
*CEP164* [62]	Centrosomal protein 164	Nephronophthisis 15; Senior–Løken syndrome; Meckel–Gruber syndrome; Joubert syndrome; Hepatic fibrosis.	11q23.3	AR	
*ANKS6* [63]	Ankyrin repeat and sterile alpha motif domain-containing 6	Nephronophthisis 16; Situs inversus; Hepatic fibrosis.	9q22.33	AR	
*IFT172* [64]	Intraflagellar transport 172	Nephronophthisis 17; Bardet–Biedl syndrome; Short-rib thoracic dysplasia 10; Hepatic fibrosis.	2p23.3	AR	Common variants in *IFT172* are associated with elevated creatine in association studies [32].
*CEP83* [65]	Centrosomal protein 83	Nephronophthisis 18; Intellectual disability; Hepatic fibrosis.	12q22	AR	
*DCDC2* [66]	Doublecortin domain-containing 2	Nephronophthisis 19; Hepatic fibrosis; Non-syndromic recessive deafness.	6q22.3	AR	
*MAPKBP1* [67]	Mitogen-activated protein kinase binding protein 1	Nephronophthisis 20	15q15.1	AR	
*IFT81* [68]	Intraflagellar transport 81	Nephronophthisis; Short-rib thoracic dysplasia 19.	12q24.11	AR	
*TRAF3IP1* [69]	TRAF3-interacting protein 1	Nephronophthisis; Senior–Løken syndrome 9; Intellectual disability.	2q37.3	AR	
*ADAMTS9* [70]	ADAM metallopeptidase with thrombospondin type 1 motif 9	Nephronophthisis	3p14.1	AR	
*INPP5E* [71]	Inositol polyphosphate-5-phosphatase E	Nephronophthisis; Joubert syndrome 1; Hepatic fibrosis; Intellectual disability.	9q34.3	AR	
*TMEM216* [72]	Transmembrane protein 216	Nephronophthisis; Joubert syndrome 2; Meckel–Gruber syndrome 2; Oro-facio-digital syndrome; Intellectual disability.	11q13.1	AR	*TTC21B* contributes possible modifier alleles [51].
*AHI1* [73]	Abelson helper-integration site 1 (Jouberin)	Nephronophthisis; Joubert syndrome 3; Intellectual disability.	6q23.3	AR	Heterozygous *AHI1* variants are enriched in patients with homozygous *NPHP1* and neurological symptoms [41]; More severe neurological disease in a patient with bi-allelic *CEP290* variants and a heterozygous *AHI1* [47]; Heterozygous *AHI1* variants are associated with retinal disease irrespective of the underlying bi-allelic cause of nephronophthisis [74].
*CC2D2A* [75]	Coiled-coil and C2 domain-containing 2A	Nephronophthisis (possible mild); Meckel–Gruber syndrome 6; Joubert syndrome 9; COACH syndrome 2; Hepatic fibrosis; Intellectual disability.	4p15.32	AR	*TTC21B* contributes possible modifier alleles [51].
*TMEM237* [76]	Transmembrane protein 237	Nephronophthisis; Joubert syndrome 14; Meckel–Gruber syndrome.	2q33.1	AR	
*TMEM138* [77]	Transmembrane protein 138	Nephronophthisis (rare); Joubert syndrome 16; Oro-facio-digital syndrome.	11q12.2	AR	
*TMEM231* [21]	Transmembrane protein 231	Cystic kidneys; Joubert syndrome 20; Oro-facio-digital syndrome 3; Meckel-Gruber syndrome 11.	16q23.1	AR	
*IFT122* [78]	Intraflagellar transport 122	Nephronophthisis; Cranio-ectodermal dysplasia 1; Hepatic fibrosis.	3q21.3-q22.1	AR	
*WDR35* [79]	WD repeat domain 35	Nephronophthisis; Cranio-ectodermal dysplasia 2; Short-rib thoracic dysplasia 7; Hepatic fibrosis.	2p24.1	AR	
*IFT43* [80]	Intraflagellar transport 43	Nephronophthisis; Cranio-ectodermal dysplasia 3; Short-rib thoracic dysplasia 18; Hepatic fibrosis.	14q24.3	AR	
*ALMS1* [81]	ALMS1 centrosome and basal-body-associated protein	Alström syndrome	2p13.1	AR	
**Autosomal-Dominant Tubulointerstitial Kidney Disease (ADTKD)**					
*UMOD* [17]	Uromodulin	ADTKD-*UMOD*	16p12.3	AD	Common promoter variants are associated with the risk of CKD and hypertension [31,82,83,84,85,86,87,88]; Bi-allelic variants are more severe [89].
*MUC1* [90]	Mucin 1, cell surface-associated	ADTKD-*MUC1*	1q22	AD	Common splice-site variant increases the risk of CKD [30].
*HNF1B* [91]	HNF1 homeobox B	ADTKD-*HNF1B*	17q12	AD	*HNF1B* has a role in transcriptional activation of *UMOD*, *PKHD1*, and *PKD2* genes [92].
*REN* [93]	Renin	ADTKD-*REN*; Renal tubular dysgenesis.	1q32.1	AD AR	Bi-allelic variants cause a more severe phenotype resulting in renal tubular dysgenesis [94].
*SEC61A1* [95]	SEC61 translocon alpha 1 subunit	ADTKD-*SEC61A1*	3q21.3	AD	
*DNAJB11* [18]	DnaJ heat-shock protein family (Hsp40) member B11	ADTKD/ADPKD “hybrid”; Ivermark II syndrome–renal-hepatic-pancreatic dysplasia (RHPD)	3q27.3	AD AR	Bi-allelic variants cause a more severe phenotype with a foetal disease, including enlarged cystic kidneys, dilation and proliferation of pancreatic duct cells, and liver ductal plate malformation [96].
**Mitochondrial disorders**					
*MT-TF* [13]	Mitochondrially encoded tRNA-Phe (UUU/C)	Mitochondrially inherited tubulointerstitial kidney disease (MITKD)	Mitochondria	Mitochondria	
*MT-TL1* [97]	Mitochondrially encoded tRNA-Leu (UUA/G) 1	Mitochondrial encephalopathy, lactic acidosis, and stroke-like episodes (MELAS); Maternally inherited diabetes and deafness (MIDD) syndromes.	Mitochondria	Mitochondria	A possible modifying variant in tRNA^lys^ in a family with m.3243A > G and MIDD rather than MELAS, the tRNA^lys^ variant was absent in 75 controls [98].
**Renal tubular dysgenesis**					
*AGT* [94]	Angiotensinogen	Renal tubular dysgenesis	1q42.2	AR	
*AGTR1* [94]	Angiotensin II receptor type 1	Renal tubular dysgenesis	3q24	AR	
*ACE* [94]	Angiotensin I converting enzyme	Renal tubular dysgenesis	17q23.3	AR	
**Other**					
*XPNPEP3* [14]	X-prolyl aminopeptidase 3	Nephronophthisis-like nephropathy 1 (NPHPL1)	22q13.2	AR	
*GATM* [15]	Glycine amidinotransferase	Fanconi syndrome and IFTA	15q21.1	AD	
*SLC41A1* [99]	Solute carrier family 41 member 1	Nephronophthisis-like nephropathy	1q32.1	AR	
*FAN1* [100]	FANCD2- and FANCI-associated nuclease 1	Karyomegalic interstitial nephritis	15q13.3	AR	

AD, autosomal dominant; ADTKD, autosomal-dominant tubulointerstitial kidney disease; ADPKD, autosomal-dominant polycystic kidney disease; AR, autosomal recessive; COACH syndrome, cerebellar vermis hypo/aplasia, oligophrenia, congenital ataxia, ocular coloboma, and hepatic fibrosis; ESKD, end-stage kidney disease; FSGS, focal segmental glomerulosclerosis; MITKD, mitochondrial inherited tubulointerstitial kidney disease.

#### 1.1.1. NPHP1

In 25% of isolated nephronophthisis cases, a large genetic deletion arises from homologous recombination of genetic repeats, resulting in deletion of 290 kb and the entire *NPHP1* gene (83 kb) [64,101,102]. Wide variation in age of ESKD (pre-puberty to seventh decade) amongst patients homozygous for this deletion suggests modifier genes contribute to the variable phenotype [41,103]. Hoefele et al. identified siblings with homozygous deletions of *NPHP1*, whereby both had nephronophthisis and retinitis pigmentosa. However, one sibling also carried a rare heterozygous variant in *NPHP4* and developed ESKD aged nine (eight years earlier than her sibling) [40].

In addition to marked differences in the age of onset, patients homozygous for *NPHP1* deletions can vary dramatically in their phenotype, from isolated nephronophthisis with progressive CKD to Senior–Løken syndrome (SLSN), Bardet–Biedl syndrome (BBS), and mild forms of JBTS (Table 2) [104,105,106].

In a cohort of patients with nephronophthisis and neurological phenotypes consistent with Joubert syndrome (JBTS), half of those with variants in *NPHP1* also harboured a second variant, including heterozygous missense variants in *AHI1* and truncating variants in *CEP290* [41]. In another study, the same hypomorphic variant in *AHI1* was associated with retinal disease in 153 patients with nephronophthisis irrespective of the underlying genetic cause of nephronophthisis [74]. Functional evidence for this modifier is seen in homozygous *Nphp1* knock-out mice (Nphp−/−) crossed with heterozygous Ahi1 knockouts (Ahi1+/−) demonstrating more significant degenerative retinal lesions [74].

#### 1.1.2. NPHP3

Autosomal-recessive variants in *NPHP3* (Nephrocystin-3) were initially thought to cause adolescent- or adult-onset nephronophthisis [42]. However, recent case reports describe much earlier onset of ESKD, with some evidence for the role of genetic modifiers [107]. In addition to pathogenic compound heterozygous variants in *NPHP3*, additional heterozygous frameshift or missense variants in *NPHP4* are described in a number of families associated with earlier disease, including antenatal mortality [40,43].

#### 1.1.3. CEP290

**Figure 3:**
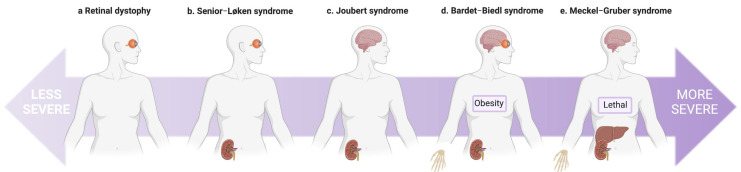
**The spectrum of disease associated with variants in centrosomal protein 290 (*CEP290*).** (**a**) Eye involvement ranges from a relatively mild retinal phenotype to Leber congenital amaurosis (LCA), which is a leading cause of childhood blindness. (**b**) Senior–Løken syndrome is characterised by retinal dystrophy and nephronophthisis. (**c**) Joubert syndrome (JBTS) adds cerebellar vermis hypoplasia to retinal dystrophy and nephronophthisis. (**d**) Bardet–Biedl syndrome is truly multisystem, with significant features of retinal disease, central obesity, postaxial polydactyly, cognitive impairment, genitourinary abnormalities, and kidney disease. (**e**) Meckel–Gruber syndrome is a lethal multisystem disorder with occipital meningo-encephalocele, hepatobiliary ductal plate malformation, postaxial polydactyly, and cystic dysplasia of the kidneys with marked interstitial fibrosis.

Autosomal-recessive variants in *CEP290* (*NPHP6*) are implicated in an extensive range of syndromic clinical ciliopathies, including LCA, SLS, JBTS, BBS, and MKS (Figure 3) and also isolated nephronophthisis [45,46,48,108,109,110]. Variant type and location do not adequately explain the highly variable phenotype [111]. A deep intronic *CEP290* variant (c.2991 + 1655A > G) resulting in aberrant splicing was identified in 16 (21%) of 76 unrelated patients with Leber congenital amaurosis (LCA). Despite altered splicing, a small amount of normally spliced protein was still present. This may explain the normal cerebellar and renal function in patients with LCA secondary to *CEP290* variants in a dosage-dependent mechanism, with complete loss of CEP290 protein function in more severe manifestations like JBTS [108]. However, differential phenotypes could equally be influenced by tissue-specific variation in gene isoform expression due to alternative splicing [112,113]. This has been previously observed for *WNK1*, where variants are alternatively expressed on different tissue-specific isoforms in the kidney and nervous system, resulting in either hyperkalaemic hypertension or hereditary sensory and autonomic neuropathy type 2, respectively [114]. Dose-dependent disease variants including alternative splicing and tissue-specific isoform expression in *CEP290* are unlikely to fully account for the observed phenotypic heterogeneity. A study screening patients with the same *CEP290* genotype but varied neurological severity revealed a novel heterozygous missense variant in *AHI1* in a more severely affected patient [47]. Furthermore, barttin CLCNK-type accessory subunit beta (*BSND*), historically associated with Bartter syndrome type 4a, has been identified to modify the severity of cystic kidney disease and renal failure progression in JBTS caused by *CEP290* variants [49]. Functional studies reveal a synergistic effect on the severity of phenotype with *CEP290* and knockout of *IQCB1* or *CC2D2A* [75,115]. Other cilia components have also been shown to genetically or physically interact to modulate CEP290 phenotypes in mouse models [116].

#### 1.1.4. RGRIP1L

Meckel–Gruber syndrome (MKS) and Bardet–Biedl syndrome (BBS) may be allelic forms of the same “molecular spectrum”, with hypomorphic variants in genes known to cause MKS (*MKS1*, *TMEM216*, and *CEP290*), also associated with BBS [48]. Bi-allelic truncating variants in *RPGRIP1L* (*NPHP8*) have been described to cause the more severe MKS compared with missense variants in the same gene causing JBTS [50,117,118,119,120,121]. In patients with a variety of syndromic ciliopathies attributed to variants in *NPHP3*, *IQCB1*, *CEP290*, and *MKS1*, a common *RPGRIP1L* variant (p.Arg229Thr) (present in over 8% of South Asians and 3% of other populations) is significantly enriched in patients who also have retinitis pigmentosa [44,122].

#### 1.1.5. TTC21B

Homozygous variants in tetratricopeptide repeat domain 21b (*TTC21B*) are associated with an array of different clinical entities including nephronophthisis, nephrotic range proteinuria, focal segmental glomerulosclerosis (FSGS), or global sclerosis [52]. Amongst a cohort of patients with a “ciliopathy”, pathogenic alleles in *TTC21B* were identified in 38/753 (5%) patients, suggesting *TTC21B* variants commonly contribute to the overall variant burden. Seventeen patients carried heterozygous variants in at least one of 13 other genes (including *NPHP4*, *RPGRIP1L*, *TMEM216*, *CC2D2A*, and *MKS1*) implicated in nephronophthisis or associated syndromes, suggesting modified genetic activity [51].

#### 1.1.6. UMOD

In addition to modifiers in other genes (in trans), a phenotype may be altered by additional variation occurring in the same gene (in cis) and on the same haplotype/chromosome as the primary disease variant (Figure 2), with exemplars seen in cystic fibrosis [123]. *UMOD* variants contribute to both monogenic TKD and polygenic forms of CKD. Common risk variants for the development of CKD and hypertension in the promoter region of *UMOD* are thought to have risen in population frequency due to selective pressure from increased urinary uromodulin defending against urinary tract infections [31,82,83,84,85,86]. A very high percentage of individuals have at least one risk variant that increases the quantity of uromodulin expressed (70 to 95%) [31,86]. These variants are highly likely to co-occur in monogenic ADTKD-*UMOD* and may explain some of the extreme variation of progression to ESKD within families with the same pathogenic variant. A study of 147 families with monogenic ADTKD-*UMOD* identified underrepresentation of a protective allele (rs4293393—associated with reduced uromodulin production) compared with large population databases. This protective allele was linked to monogenic *UMOD* variants in only 11.6% of affected families but was in cis with “wild-type” *UMOD* in 17%. This compares with an expected minor allele frequency of 18–20% from the *Genome Aggregation Database* (*gnomAD*) [26]. The authors postulate that this decreased protective allele frequency may be due to decreased expression of mutated uromodulin being less likely to receive a molecular diagnosis due to a milder clinical phenotype (with later development of ESKD) [73]. Rare cases of homozygous *UMOD* variants in two consanguineous families demonstrate a more severe phenotype than with heterozygous variants [89,124].

#### 1.1.7. MUC1

ADTKD-MUC1 frequently results from the insertion of an additional cytosine into a variable number of tandem repeats (VNTRs), resulting in a frameshift variant in *MUC1* [90,122]. The high-guanosine/cytosine (GC) content of the VNTR region hinders short read sequencing, and therefore, ADTKD-*MUC1* is probably underdiagnosed. The age of onset of ESKD ranges from 16 to 80 years [1]. With an allele frequency of 42% (*gnomAD* genomes), a common variant in *MUC1* (rs4072037) is likely to coexist with monogenic forms of ADTKD-*MUC1*. It is an attractive genetic modifier candidate, as it influences gene expression through alternative splice-site mechanisms and is associated with declining kidney function in GWAS [30].

#### 1.1.8. HNF1B

**Figure 4:**
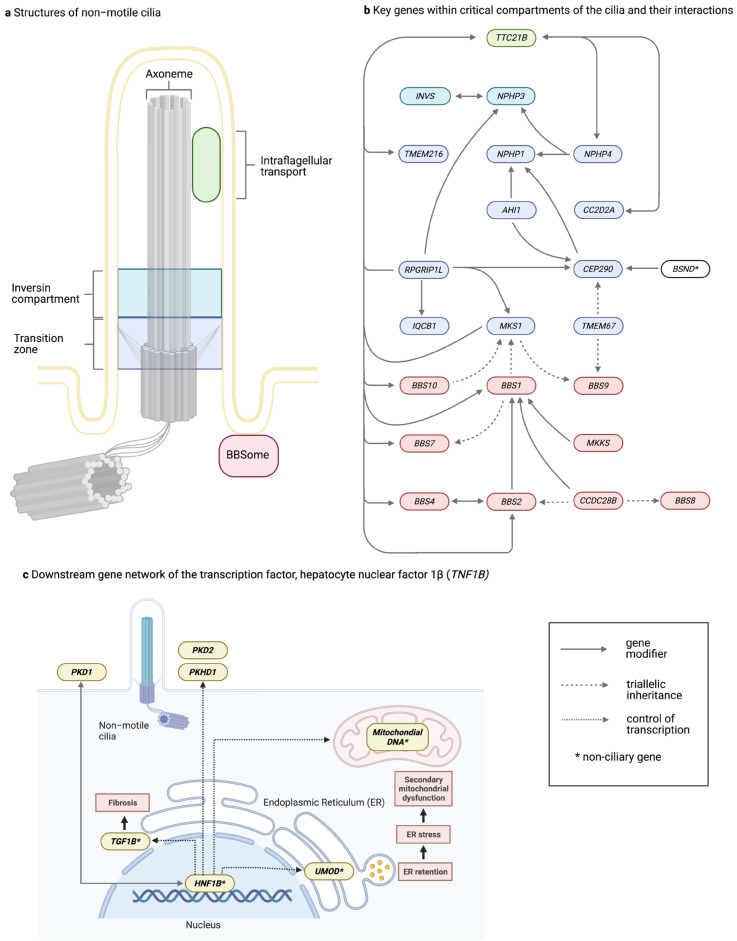
Gene regulatory networks in tubulointerstitial kidney disease with evidence of modifier genes, tri-allelic inheritance, or gene interaction. (**a**) Non-motile (primary) cilia dysfunction is a recognised driver of kidney disease, including nephronophthisis, polycystic kidney disease, or cystic dysplasia (Figure 1). Cilia are membrane-bound organelles that resemble finger-like projections on the apical surface of many tissues, including renal tubular epithelia. The primary cilium is composed of a basal body consisting of triplets of microtubules (grey) from which the cilium assembles [147]. (**b**) Hotspots for ciliopathies include the intraflagellar transport proteins (green) responsible for protein trafficking and signalling pathways, the inversin compartment (blue) controlling cell polarity, the transition zone (purple) involved in the cell cycle and control of the entry and exit of proteins, and the BBSome (Bardet–Biedl syndrome proteins) (red) responsible for the trafficking of proteins to the cilia [147]. This network of selected genes with evidence of modifier and tri-allelic inheritance reveals the close functional interaction of genes encoding the primary cilia. Solid lines represent modifier genes, and dashed lines represent genes with evidence for possible tri-allelic inheritance. The direction of the arrow denotes the direction of the contribution of additional variants to the primary mutated gene (e.g., homozygous variants in *NPHP1* and a third mutated allele in *AHI1* may result in a neurological phenotype like Joubert syndrome) [41]. Bidirectional arrows indicate the equal contribution of variants. (**c**) There is significant potential for cumulative variantal burden in genes downstream of the transcription factor hepatocyte nuclear factor 1β (*HNF1B*) to modify disease phenotypes. Evidence in mouse models suggests that several genes are downregulated with *Hnf1b* inactivation (dashed line) [92]. Downregulation of *UMOD* may also explain why most patients with *HNF1B* variants have hyperuricaemia typical of ADTKD-*UMOD* [148]. Loss of *HNF1B* results in activation of a transcriptional network that induces extracellular matrix deposition and aberrant transforming growth factor 1 beta (*TGF1B*) signalling, resulting in tubulointerstitial fibrosis [149]. *HNF1B* is linked to mitochondrial dysfunction in renal epithelial cells in experimental studies through other transcription factors [150]. Mitochondrial dysfunction in ADTKD-*UMOD* also results secondary to endoplasmic reticulum stress from retention of misfolded uromodulin protein [133]. Direct evidence of genetic modification has been reported in a patient with heterozygous variants in both *PKD1* and *HNF1B*, causing early-onset severe disease [130].

Heterozygous variants in *HNF1B* or 17q12 microdeletions encompassing the *HNF1B* gene are associated with large intra-familial variation in TKD (with and without cysts), maturity-onset diabetes of the young type 5 (MODY5), CAKUT, and other organ involvement, including neuropsychiatric symptoms [125]. Structural kidney abnormalities have been significantly associated with splice-site variants and MODY5, specifically with frameshift variants [126]. However, an extensive retrospective analysis of 377 patients with *HNF1B* kidney disease revealed no correlation between the genetic variant and renal failure severity [127]. Although the co-occurrence of neurodevelopmental disorders may be due to other genes in the microdeletion, more recent data suggest a role for epigenetic modifiers due to differential methylation patterns [128,129]. Evidence for the role of genetic modifiers is emerging, with heterozygous *HNF1B* variants detected with heterozygous *PKD1* variants in a patient with early and more severe polycystic kidney disease [130]. Another possible explanation for the wide phenotypic variability could be the influence of *HNF1B* as a transcription factor on the transcription of multiple other genes and, therefore, the specific variant burden in each of these downstream genes (Figure 4).

#### 1.1.9. Mitochondrial Function

There are hundreds of mitochondrial DNA copies in each cell, and therefore, mutated copies may exist with normal copies in a state known as heteroplasmy. Disease expression depends on the proportion of dysfunctional mitochondria and tissue distribution [131]. Multiple organs may be affected, including the kidneys, skeletal muscle, and central nervous system. The range of kidney diseases associated with a m.3243A > G variant in the tRNA^Leu^ gene includes TKD and isolated tubulopathies, cystic kidney diseases, FSGS, as well as syndromic forms, including MELAS (myopathy, encephalopathy, lactic acidosis, and stroke-like episodes) and maternally inherited diabetes and deafness (MIDD) syndrome [98,132]. One family with MIDD syndrome also carried a second variant in tRNA^lys^, which was absent in 75 controls. Isolated TKD phenotypes also occur in cases where all copies of (homoplasmic) mitochondrial DNA are mutated [13].

Secondary mitochondrial dysfunction also occurs in ADTKD-*UMOD*, resulting from the unfolded protein response and increased ER stress, leading to a decrease in the number of mitochondria and associated proteins [133]. Any additional modifiers in the mitochondrial genome could affect the phenotype further (Figure 4).

### 1.2. Evidence for Oligogenic Inheritance

Bardet–Biedl syndrome (BBS) is a multisystem disorder underpinned mainly by dysfunction of primary cilia (Table 1 and Figure 4). Renal involvement in BBS is highly variable, including urine retention due to aberrant water trafficking, TKD, cystic dysplasia, hydronephrosis, and CAKUT. Despite wide renal phenotypic heterogeneity, no genotype correlation has been identified [134]. A multi-protein complex called the BBSome comprises protein subunits encoded by BBS genes. BBS was initially thought to be a monogenic recessive disorder; however, deviation from the expected autosomal-recessive inheritance pattern in many pedigrees suggested alternative inheritance models. Following the screening of a large cohort of BBS families for variants in *BBS2* and *MKKS*, tri-allelic inheritance was demonstrated in affected individuals from four pedigrees and revealed unaffected individuals from two pedigrees that carried two *BBS2* variants but none in *MKKS* [135]. This is an example of digenic, tri-allelic inheritance (Figure 2). Since then, tri-allelic inheritance has been identified in other cohorts of BBS patients and other diseases [37,136,137,138]. Beales et al. analysed 259 families and found evidence of tri-allelic inheritance and a homozygous missense variant of p.Met390Arg (the most frequent *BBS1* variant in European populations) in asymptomatic members of two families, suggesting either incomplete penetrance or tri-allelic inheritance [136]. Other studies have failed to identify tri-allelic inheritance in affected cases or have identified unaffected cases with bi-allelic inheritance [137,138,139,140,141,142,143] In other pedigrees, disease was identified in family members with rare double-homozygous *BBS2* and BBS4 variants but not in tri-allelic first-degree relatives [48,144].

In a recent systematic secondary-variant burden analysis of patients with known bi-allelic variants in 1 of 17 BBS genes, researchers observed, in two independent cohorts, a non-random twofold enrichment of ultra-rare variants in other BBS genes compared with other recessive alleles and population controls [145]. The suppression of 19 gene pairs in zebrafish revealed additive or suppressive effects and significant over-representation of secondary variants in BBS complex chaperonin genes.

Although most reports of BBS involve rare variants, functional evidence exists of a common modifier (1.4% of controls) in *CCDC28B* to disease severity [137]. Given its population frequency, it may have been missed by traditional variant-filtration approaches.

The relatively high incidence of renal developmental abnormalities and renal cell carcinoma in BBS patients’ relatives may represent heterozygous carriers [146]. These findings suggest that tri-allelic inheritance results from modifier genes’ action rather than oligogenic inheritance per se and that the degree of penetrance or expressivity is related to the cumulative effect of the variants.

## 2. Discussion

The historical dichotomy of TKD as either monogenic or polygenic is overly simplistic. Evidence strongly suggests that TKD can be impacted by a diverse set of modifiers as opposed to the simpler traditional model of one-gene, one-phenotype [37,135,136,137,138,139,140,141,142,143,144,146,151,152]. The high prevalence of disease-specific modifier variants suggests their broad role in TKD and even CKD of all causes [32,44,137]. Common risk variants may prove significant when present in the same monogenic disease gene, for example, when in cis with primary pathogenic variants of *UMOD* and *MUC1* [30,31].

Monogenic disease suffers from the “Winner’s curse”, leading to a curtailing of genetic investigation once a single variant is identified. However, penetrance and expressivity of TKD are likely to be governed by the total variant burden both within individual genes and across multiple gene pathways involved in the structure and function of the tubulointerstitium. The pleiotropic effects of pathogenic variants in *HNF1B* demonstrate the potential impact of gene networks [119,153]. Multiple genetic “hits” in these TKD genes are likely to contribute variably to the disease spectrum, from apparent monogenic to polygenic or multifactorial disease patterns. Here, we describe severe rare modifiers and common moderate modifiers. However, it is essential to consider the role of multiple small-effect variants traditionally associated with polygenic diseases whose overall burden may be cumulatively large in some individuals yet seldom identified due to weak association. Most studies focus on modifiers that result in more severe diseases; however, patients with milder phenotypes who are less likely to receive a molecular diagnosis are equally likely to be subject to modifier genes. Only a minority of patients with TKD have overt extra-renal or syndromic manifestations, and even the renal phenotype may be subtle. With bland urinalysis or occasionally mild proteinuria and an association with renal cysts that is neither universal nor pathognomonic, TKD is difficult to differentiate from other non-proteinuric CKD [6,154,155].

Several barriers remain to understanding the more complex genetics of TKD, including poor differentiation of TKD from other causes of CKD, inadequate phenotyping and nomenclature, and methodological challenges, and biases in genetic analyses.

Recent evidence has identified relatively “mild”, adult-onset disease with recessive inheritance and, conversely, severe paediatric-onset disease resulting from dominant gene variants. For example, autosomal-recessive nephronophthisis due to *NPHP1*, the commonest genetic cause of paediatric ESKD, has recently been implicated in at least 0.5% of adult-onset ESKD [9,16,103]. Conversely, autosomal-dominant TKD resulting from variants in *HNF1B* and *REN* can cause severe disease in childhood [127,156]. Molecular genetics has exposed the limitations of traditional clinical diagnostics not only due to incomplete phenotyping but with phenocopies increasingly identified following molecular genetic testing [157,158]. Attempts to increase diagnoses have led to multiple and sometimes misleading nomenclature that in some cases has been used to predefine limited molecular diagnoses, such as the use of ADTKD-NOS (not otherwise specified).

Multiple and sometimes misleading disease names predefine limited molecular diagnostics and reinforce bias (Figure 1). Attempts to increase diagnoses have led to terms such as autosomal-dominant tubulointerstitial kidney disease (ADTKD) even before a molecular diagnosis has been made [6].

Although massive datasets of normal variant distribution are now available, these allow comparison of the burden of rare variants on a population level but not on an individual level and often fail to provide contextual information for multiple variants. Additionally, extensive genomic datasets have historically been biased towards white European ancestry.

GWAS data implicate coding and non-coding variants with a variety of kidney traits, and these may prove important in the polygenic inheritance patterns of TKD. The challenge facing the research community is in the use of these data to identify variants that may contribute to the risk of disease progression or the emergence of TKD. One approach may be utilising resources that map expression quantitative trait loci (eQTLs) across multiple human tissues, aiding in the prioritisation of disease-causing genes [159].

Genetic modifiers are methodologically challenging to detect and expensive to prove functionally. Classifying variants as benign or pathogenic requires multiple types of evidence [160]. The challenges to functional modelling of genetic variants in model systems for single-variant disorders scale exponentially with additional component alleles. The more alleles required to be modelled, the greater the experimental design’s time, cost, and complexity.

Improved definition of the molecular mechanisms underlying TKD is required to inform prognosis and shift the emphasis from reactive therapies to secondary or primary prevention. Extensive prospective NGS analyses have the potential to unravel the genetic and phenotypic complexities of so-called monogenic kidney diseases, not least of all TKD.

## 3. Key Points

- Tubulointerstitial kidney disease may present with a severe syndromic phenotype traditionally diagnosed in childhood, yet most TKD patients manifest a subtle phenotype with little to differentiate them from other common forms of non-proteinuric chronic kidney disease.
- Phenotypes may vary even within families that share the same putative monogenic gene variant and shared environmental factors.
- Common variants in monogenic TKD genes are also associated with chronic kidney disease at the population level.
- There is growing evidence for tri-allelic inheritance as well as for rare modifiers of severe effect and common modifiers of moderate effect on patient phenotypes.

## Figures and Tables

**Table 1:** The clinical entities and phenotypic descriptors constituting tubulointerstitial kidney diseases (TKD).

Disease	Phenotype	Genes (Alias Symbols in Parentheses)
**Autosomal-dominant tubulointerstitial kidney disease (ADTKD-*UMOD*)** Also known as: uromodulin-associated kidney disease (UAKD), familial juvenile hyperuricaemic nephropathy (FJHN), medullary cystic kidney disease type 2 (MCKD2)	Variably progressive CKD with IF/TA and minimal to no proteinuria; Early-onset hyperuricaemia/gout.	*UMOD*
**Autosomal -dominant tubulointerstitial kidney disease (ADTKD-*MUC1*)** Also known as: mucin-1 kidney disease (MKD), medullary cystic kidney disease type 1 (MCKD1)	Variably progressive CKD with IF/TA and minimal to no proteinuria.	*MUC1*
**Autosomal-dominant tubulointerstitial kidney Disease (ADTKD-*REN*)** Also known as: familial juvenile hyperuricaemic nephropathy type 2 (FJHN2)	Variably progressive CKD with IF/TA and minimal to no proteinuria; Childhood/adolescent onset: anaemia, hyperkalaemia, acidosis, progressive CKD, and development of gout; Adult-onset: slowly progressive CKD from the third decade, with or without gout.	*REN*
**Autosomal-dominant tubulointerstitial kidney disease (ADTKD-*HNF1B*)**	Associated features are variable and include: Variably progressive CKD with IF/TA and minimal to no proteinuria; Congenital anomalies of kidney and urinary tract (CAKUT); RCAD (Renal Cyst and Diabetes Syndrome); Pancreatic hypoplasia; MODY5 (Maturity-Onset Diabetes mellitus of the Young type 5); Urogenital malformations; Hypomagnesaemia; Cognitive impairment/autism spectrum disorder (associated with 17q12 deletion).	*HNF1B*
**Autosomal-dominant tubulointerstitial kidney disease (ADTKD-*SEC61A1*)**	Variably progressive CKD with IF/TA and minimal to no proteinuria; Small dysplastic kidneys; Congenital anaemia and neutropenia (with recurrent cutaneous abscesses); Growth retardation.	*SEC61A1*
**Autosomal-dominant tubulointerstitial kidney disease/autosomal-dominant polycystic kidney disease (ADTKD/ADPKD) overlap**	Variably progressive CKD and hypertension; Non-enlarged cystic kidneys with interstitial fibrosis progressing to renal atrophy; Gout.	*DNAJB11*
**Nephronophthisis (NPHP)**	Impaired urinary concentrating ability and sodium reabsorption (polyuria, polydipsia); Normal or slightly small kidneys with increased echogenicity; Variably progressive CKD with IF/TA and minimal to no proteinuria; (Hepatic fibrosis, Situs inversus); Sometimes categorised by median age of onset: - Infantile (by 1 year of age); - Juvenile (by 13 years of age); - Adolescent (by 19 years of age); - Adult.	*NPHP1*, *INVS* ^†^ (*NPHP2*), *NPHP3* ^†^, *NPHP4* ^†^, *IQCB1* (*NPHP5*), *CEP290* ^†^ (*NPHP6*), *GLIS2* (*NPHP7*), *RGRIP1L* ^†^ (*NPHP8*), *NEK8* (*NPHP9*), *SDCCAG8* (*NPHP10*), *TMEM67* ^†^ (*NPHP11*), *TTC21B* ^†^ (*NPHP12*), *WDR19* (*NPHP13*), *ZNF423* (*NPHP14*), *CEP164* (*NPHP15*), *ANKS6* (*NPHP16*), *IFT172* (*NPHP17*), *CEP83* (*NPHP18*), *DCDC2* (*NPHP19*), *MAPKBP1* (*NPHP20*), *IFT81*, *TRAF3IP1*, *ADMATS9*, *INPP5E*, *TMEM216*, *AHI1* ^†^, *CC2D2A*, *TMEM237*, *TMEM138*, *IFT122*, *WDR35*, *IFT43*.
**Senior–Løken syndrome (SLSN)**	Nephronophthisis; Retinitis pigmentosa, Leber congenital amaurosis (LCA); (Hepatic fibrosis, situs inversus).	*NPHP1* (*SLSN1*), *NPHP3* ^†^ (*SLSN3*), *NPHP4* ^†^ (*SLSN4*), *IQCB1* (*SLSN5*, *NPHP5*), *CEP290* ^†^ (*SLSN6*), *SDCCAG8* (*SLSN7*), *WDR19* (*SLSN8*), *CEP164*, *TRAF3IP1* (*SLSN9*).
**Joubert syndrome (JBTS)**	Nephronophthisis; Renal cystic dysplasia; Cerebellar vermis hypoplasia (characteristic “molar tooth” sign on MRI brain); Ataxia, hypotonia; Hepatic fibrosis; Situs inversus; Polydactyly; Intellectual disability.	*INPP5E* (*JBTS1*), *TMEM216* (*JBTS2*), *AHI1* ^†^ (*JBTS3*), *NPHP1* (*JBTS4*), *CEP290* ^†^ (*JBTS5*), *TMEM67* ^†^ (*JBTS6*), *RPGRIP1L* ^†^ (*JBTS7*), *ARL13B* * (*JBTS8*), *CC2D2A* (*JBTS9*), *OFD1* (*JBTS10*), *TTC21B* ^†^ (*JBTS11/NPHP12*), *KIF7* * (*JBTS12*), *TCTN1* * (*JBTS13*), *TMEM237* (*JBTS14*), *CEP41* * (*JBTS15*), *TMEM138* (*JBTS16*), *CPLANE1* * (*JBTS17*), *TCTN3* (*JBTS18*), *ZNF423* (*JBTS19*), *TMEM231* (*JBTS20*), *CSPP1* (*JBTS21*), *PDE6D* (*JBTS22*), *KIAA0586* * (*JBTS23*), TCTN2 * (*JBTS24*), *CEP104* * (*JBTS25*), *KIAA0556* * (*JBTS26*), *B9D1* (*JBTS27*), *MKS1* (*JBTS28*), *TMEM107* (*JBTS29*), *ARMC9* * (*JBTS30*), *CEP120* (*JBTS31*), *SUFU* (*JBTS32*), *PIBF1* (*JBTS33*), *B9D2* (*JBTS34*), *ARL3* (*JBTS35*), *BSND* ^≠^.
**Meckel–Gruber syndrome (MKS)**	Enlarged dysplastic cystic kidneys; Occipital encephalocele; Cleft palate; Hepatic fibrosis; Variable: polydactyly, skeletal dysplasia, and situs inversus.	*MKS1*, *TMEM216* (*MKS2*), *TMEM67* ^†^ (*MKS3*), *CEP290* ^†^ (*MKS4*), *RPGRIP1L* ^†^ (*MKS5*), *CC2D2A* (*MKS6*), *NPHP3* ^†^ (*MKS7*), *TCTN2* * (*MKS8*), *B9D1* (*MKS9*), *B9D2* (*MKS10*), *TMEM231* (*MKS11*), *KIF14* (*MKS12*), *TMEM107* (*MKS13*), *CSPP1*, *TXNDC15*, *TMEM237*, *CPLANE1* *, *CEP55*.
**COACH syndrome**	Nephronophthisis; Renal cystic dysplasia; Cerebellar vermis hypoplasia, oligophrenia, ataxia, coloboma, and hepatic fibrosis.	*TMEM67* ^†^, *CC2D2A*, *RPGRIP1L* ^†^.
**Short-rib thoracic dysplasia (SRTD) with or without polydactyly** Also known as: asphyxiating thoracic dystrophy; Jeune syndrome	Nephronophthisis; Renal cystic dysplasia; Constricted thoracic cage; Short ribs; Shortened tubular bones; Variable: multiorgan involvement, polydactyly, hepatic fibrosis, and intellectual disability.	*CEP120*, *CSPP1*, *DYNC2H1*, *DYNC2LI1*, *IFT140*, *IFT172*, *IFT43*, *IFT52*, *IFT80*, *IFT81*, *INTU*, *KIAA0586* *, *NEK1*, *TCTEX1D2*, *TTC21B* ^†^, *WDR19*, *WDR34*, *WDR35*, *WDR60*.
**Cranio-ectodermal dysplasia (CED)**	Nephronophthisis; Skeletal abnormalities; Craniosynostosis (premature closure of cranial sutures); Ectodermal abnormalities.	*IFT122* (*CED1*), *WDR35* (*CED2*), *IFT43* (*CED3*), *WDR19* (*NPHP13*, *CED4*).
**Bardet–Biedl Syndrome (BBS)**	Nephronophthisis; Renal cystic dysplasia; Focal segmental glomerulosclerosis (FSGS); Rod-cone dystrophy; Polydactyly; Obesity; Genital malformations; Intellectual disability.	*BBS1*, *BBS2*, *ARL6* (*BBS3*), *BBS4*, *BBS5*, *MKKS* (*BBS6*), *BBS7*, *TTC8* (*BBS8*), *BBS9*, *BBS10*, *TRIM32* (*BBS11*), *BBS12*, *MKS1* ^†^ (*BBS13*), *CEP290* ^†^ (*BBS14*), *WDPCP* ^†^ (*BBS15*), *SDCCAG8* (*BBS16*), *LZTFL1* (*BBS17*), *BBIP1* (*BBS18*), *IFT27* (*BBS19*), *IFT74* (*BBS20*), *C8ORF37* (*BBS21*), *IFT172*, *NPHP1*, *CCDC28B* ^≠^, *TMEM67* ^†^.
**Alström syndrome**	Progressive CKD with IF/TA; Cone-rod dystrophy; Obesity; Progressive sensorineural hearing loss; Cardiomyopathy; Type 2 diabetes.	*ALMS1*
**Karyomegalic Interstitial Nephritis (KIN)**	Variably progressive CKD; IF/TA with enlarged and atypical tubular epithelial cell nuclei.	*FAN1*
**Renal tubular dysgenesis**	Foetal anuria and perinatal death from pulmonary hypoplasia and oligohydramnios (Potter syndrome).	*REN*, *AGT*, *AGTR1*, *ACE*.
**Mitochondrial Inherited Tubulointerstitial Kidney Disease (MITKD)**	Isolated variably progressive CKD with bland urinalysis and IF/TA and no Fanconi syndrome or extra-renal manifestations; Tubulopathy, including Fanconi syndrome; FSGS, glomerulocystic kidney disease; A multisystem disease of muscles and neurological system but can include other organ systems: Mitochondrial encephalopathy, lactic acidosis, and stroke-like episodes (MELAS); Maternally inherited diabetes and deafness (MIDD) syndrome; Mitochondrial DNA deletion syndromes.	*MTTF* (mt-tRNA^Phe^), *ML-TL1* (mt-tRNA^Leu^), mitochondrial DNA deletions.

* No convincing evidence of renal disease, ^†^ “causative” and disease modifier, ^≠^ disease modifier only.

## Data Availability

No new data was curated for this review article.
